# The relationship between social support and participation in stroke: A systematic review

**DOI:** 10.4102/ajod.v7i0.357

**Published:** 2018-10-10

**Authors:** Toughieda Elloker, Anthea J. Rhoda

**Affiliations:** 1 Department of Physiotherapy, University of the Western Cape, South Africa; 2 Faculty of Community and Health Sciences, University of the Western Cape, South Africa

## Abstract

**Background:**

The incidence of cerebrovascular accidents with its devastating effects on individuals is increasing. Post-stroke, restrictions in participation are common and social support could have an influence on this. Social support provided to individuals post-stroke is vital, but the relationship between social support and participation is not well understood.

**Objectives:**

This review aimed to systematically determine the relationship between social support and participation post-stroke, based on the literature available.

**Method:**

Ebscohost, Science Direct, Biomed Central, Cochrane Library, Google Scholar, Pedro Central and Wiley Online were the electronic databases searched between 2001 and 2016. Articles were deemed to be eligible if they met the inclusion criteria and successfully underwent scrutiny to determine their relevance and methodological quality, using tools from the Critical Appraisal Skills Programme and Milton Keynes Primary Trust. A narrative synthesis method was used to analyse the included studies.

**Results:**

A total of 54 articles were identified after screening, and six articles were deemed eligible for inclusion. The articles consisted of cross-sectional, qualitative and cohort studies. Articles showed distinct, significant relationships between social support and participation where the quality and quantity of social support were important. High levels of social support had a positive influence on participation, social and leisure activities, as well as returning to work post-stroke.

**Conclusion:**

A positive relationship exists between social support and participation post-stroke. Health professionals need to include social support interventions when attempting to manage the individual with stroke holistically, as this will have positive effects on participation.

## Introduction

Cerebrovascular accidents or stroke remain a leading cause of death and disability in South Africa (Bryer et al. 2011) and the incidence is increasing. After suffering from a stroke, the body structures and functions become impaired and, as a result, the individual might experience difficulties in performing basic activities of daily living (ADLs). Restrictions in participation have also been reported (Maleka et al. 2012; Rouillard et al. 2012; Rhoda et al. 2015), regardless of stroke severity (Wolf & Koster 2013). Participation is a concept defined as an individual’s involvement in life situations which include meaningful activity, community, family, work, social and civic life (World Health Organization 2001), and restrictions in these domains have been documented (Wolf & Koster 2013). According to a recent study, two fundamental principles of participation include social engagement (with family and friends) and aspects of self-care (activities to maintain health) (Resnik et al. 2012). Although these factors have been identified as principles of participation, they have also been found to influence participation (Geyh et al. 2004; Wolf & Koster 2013). These facets are further classified within the International Classification of Functioning, Disability and Health (ICF) as environmental factors and activity limitations, respectively (WHO 2001). This framework is directed at reflecting the dynamic collaboration between the domains of activity, participation and environmental factors such as social support, while describing participation as being influenced by them (Fallahpour et al. 2011).

As a result of the impairment following stroke, some individuals may be unable to return to their pre-stroke activities and roles, and often have to depend on friends and family for support. This can become challenging, as these relationships are often adversely affected (O’Sullivan & Chard 2010).

In the attempt to support individuals with stroke to return to their previous functioning, it is necessary to consider the social support structures available to them. The term social support has been considered in studies of health and well-being since the early 1970s (Tsouna-Hadjis et al. 2000), and is defined as ‘the availability or provision of a relationship, information or assistance that empower a person to manage their day to day life effectively in the presence or absence of crisis’ (Newsham 1998, cited in Beckley 2006:126). Known as a multi-faceted concept, social support can be categorised into three different elements (Fallatah & Edge 2015). Emotional support refers to caring, acceptance and listening, instrumental support entails practical help from some other person, while informational support includes the provision of knowledge to help solve practical problems (Wills & Shinar 2000). Collectively, these elements can be referred to as the quality of social support (Glass & Maddox 1992). The number of persons in a support network and the amount of time invested by this network, as well as the frequency of availability of social support, is defined as the quantity of social support (Glass et al. 1993; Tsouna-Hadjis et al. 2000). While all three types of support were shown to improve function (Glass & Maddox 1992), a high level of instrumental support has a positive impact on social (social involvement) and functional status (ADLs), while a high magnitude of emotional support has a profound effect on patients’ psychosocial health (depression) (Tsouna-Hadjis et al. 2000). In addition, a large amount of social support has been shown to provide a quicker and more extensive recovery of function in ADLs (Glass et al. 1993).

The effects of social support on improved functional recovery and psychosocial health are clearly outlined in the literature above. With regard to participation, Beckley (2006) found that social support moderates the effect of functional limitations on participation. The evidence for social support stems from the study’s conclusion that improvements in both functional limitation and participation restrictions are dependent on social support. The study findings stress that the levels of subjective social support result in improved functional status. The level of subjective social support could reflect the amounts of support reported by participants. This, in turn, can improve participation. This study did not directly measure social support and its influence on participation. If participation was included as an outcome measure, the conclusion with regard to the relationship between social support and participation would be better understood. There is existing literature that directly examines the relationship between social support and participation (Mayo et al. 2013). However, this literature is minimal, especially in the developed world. The purpose of conducting this review was to discover all studies that show a direct link between the two variables. The evidence for this relationship has not been systematically presented and, as a result, this relationship is not clearly understood, which explains the rationale for this review. This review aims to systematically identify the relationship between social support and participation in individuals living with stroke. The research question this review intends to answer is: In community-dwelling individuals with stroke, what is the relationship between social support and participation post-stroke?

## Methodology

A systematic approach to conducting the review was adopted. This review is in compliance with the preferred reporting items for systematic reviews and meta-analysis (PRISMA) guidelines (Moher et al. 2009) and was also registered with PROSPERO (registration number: CRD42018086142). The online supplementary material can be accessed at http://www.crd.york.ac.uk/PROSPERO/displayrecord.php?ID=CRD42018086142.

### Search strategy

The databases of Ebscohost full-text, which included CINAHL +, Health Source: Nursing, Academic edition, Medline, Psych articles and Soc. index, Science Direct, Biomed Central, Google Scholar, Cochrane Library, Pedro Central and Wiley Online, were searched to access articles published between January 2001 and October 2016. These databases were accessed from the University Library, under the advice and supervision of an expert Librarian. The year 2001 was chosen as a starting point as it coincides with the publication of the revised International Classification of Impairment, Disability and Handicap (ICIDH). In the ICF, the concept of participation could be seen to replace handicap, and includes the influence of contextual factors on disability. The same key search terms were used for all databases with Boolean operators such as ‘AND’ and ‘OR’. The electronic search was conducted using the PubMed search builder. The key terms used were social support AND (participation OR participation restrictions) AND (stroke OR CVA) AND (recovery OR rehabilitation). The same approach was used for all searches but was adapted as necessary according to the database. Medical Subject Headings (MeSH) terms were used in databases that made use of that function. Search limiters were applied to include only full-text, English articles, published in peer-reviewed journals on human subjects, published in the years of interest.

### Eligibility criteria

Articles were deemed to be eligible if they met the inclusion criteria, successfully underwent scrutiny via the population, intervention, comparison and outcome (PICO) method, and obtained a moderate score (see Appendix 1) for their quality assessment.

The following inclusion criteria were used:

1. individuals with a primary diagnosis of stroke
2. individuals with stroke who were community dwelling
3. studies that measured at least one domain of participation as identified by the ICF, and one dimension of social support
4. studies that used the ICF as a framework to link participation restrictions and environmental factors such as social support
5. any article, the outcomes of which measured both participation and social support, not necessarily measuring the relationship between the two
6. any study designs
7. intervention-based studies where a social support intervention is compared with normal care
8. availability of the English full-text version of the publication
9. articles published in a peer-reviewed journal.

Articles were excluded if the stated criteria were not met.

#### Population, intervention, comparison and outcome

Articles were screened initially by reviewing titles and abstracts. Selected articles then underwent review using the PICO method. The term PICO is described as population, intervention, comparison and outcome (Appendix 1). The relevance of the articles during the PICO process was reviewed by two independent reviewers. Where consensus was not reached, reviewers discussed the differences in opinion and came to a unanimous decision. The articles that were found relevant for inclusion, following analysis via the PICO method, were then subject to undergoing the methodological quality assessment.

#### Quality assessment

The Critical Appraisal Skills Programme (CASP 1994) and Milton Keynes Primary Trust (2002) were the tools used to assess the articles’ methodological quality which includes a risk of bias assessment. This was conducted by two independent reviewers who were required to score each article. Each tool consisted of 10–12 questions, two of which were screening questions that did not impact the final scoring. The remaining questions were more detailed and had guidelines for the authors to assess the questions critically. Both tools assessed each article in terms of sampling, outcome measures, data collection procedure, analysis of data, precision of results and study findings. More specifically, the risk of bias was determined by assessing whether the outcome was measured subjectively or objectively, and if it had been validated. The rigour of the methodology was assessed by looking at the setting for data collection, whether the data collection methods were clear, if the researcher had justified the methods and whether the methods were explained explicitly. Appendix 2 is an example of the CASP cross-sectional tool used to measure the quality assessment of the cross-sectional studies. Articles that scored between 8 and 10/10 were viewed as having a high score, 5 and 7/10 a moderate score and 1 and 4/10 a poor score (Kumerenzi et al. 2010). The articles that scored five and above out of 10 were included in this review.

### The data extraction tool

A data extraction tool was developed based on the literature from Kumerenzi et al. (2010). The data gathered from the extraction tool included but were not limited to: Author(s) name(s), country, participant demographic details, study design, data collection instrument, outcomes measured and the results of the study.

### Data analysis

A narrative synthesis was used to analyse the data obtained from the included studies. This method of data analysis is usually used to synthesise data gathered from a wide range of study designs, which rely on the use of words and texts to explain and summarise findings (Popay et al. 2006). This process includes developing a theory for how the interventions work, examining the study findings systematically, exploring relationships in the data between studies and assessing the amount and quality of the evidence (Ryan 2013).

## Results

**FIGURE 1:**
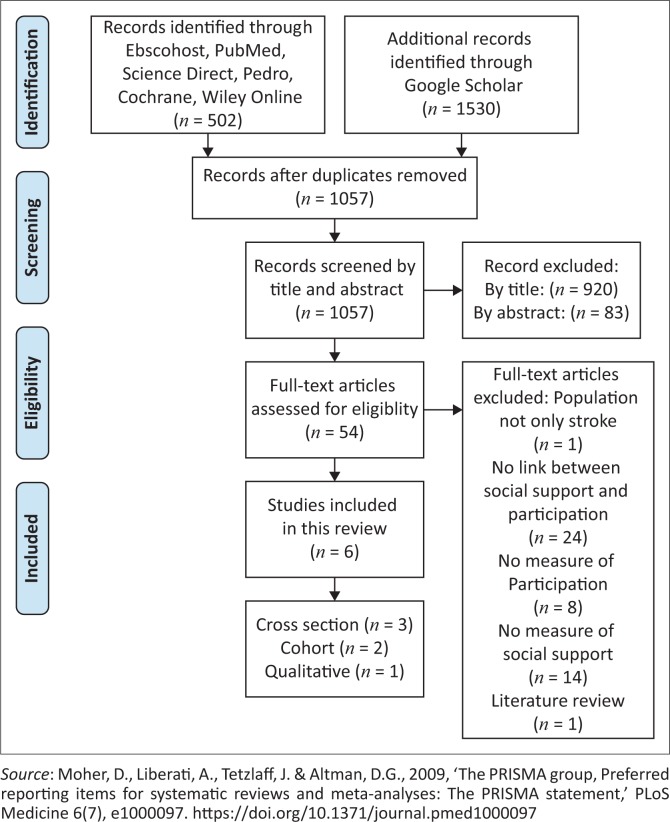
Flow diagram of study selection.

A total of 502 articles were generated from the databases from the first hit of the key terms and the MeSH terms. Google Scholar was accessed to identify grey literature and generated a further 1530 hits. Following the application of the inclusion criteria to the titles, 1057 duplicates were removed and 920 articles were excluded. A further 83 articles were excluded after screening abstracts. To determine the eligibility of the remaining 54 studies, the PICO method and inclusion criteria were applied to each article. No randomised control trials (RCTs) were identified, so all articles had no intervention and comparison groups. After the two assessors conducted the PICO and quality assessment, a total of six articles were included. The reasons for excluding the 48 articles are presented in Figure 1, along with the study selection. Matters discussed amongst assessors included articles which measured participation and included aspects of social support, although dimensions of social support were not measured. The term social participation in relation to social support was also discussed. The use of an independent third party was not necessary, as the two primary assessors were able to reach consensus regarding all articles.

### Characteristics of included studies

Beckley (2007) and Vincent-Onabajo et al. (2016) reported on social support and its effect on participation, while Choi et al. (2015) conducted a path analysis to determine psychosocial predictors of participation restrictions post-stroke. Two cohorts were identified by Mayo et al. (2013) and Norlander et al. (2016). Mayo et al. (2013) assessed participation and its influence on walking capacity, mood and social support post-stroke and Norlander et al. (2016) identified factors that predict social and leisure activities at 16 months and 10 years post-stroke onset. The qualitative study by Sumathipala et al. (2011) reported on how contextual factors identified by the ICF influenced long-term needs after stroke. Table 1 provides more information on these articles.

**TABLE 1:** Articles that were reviewed and met the criteria for the study.

Authors	Country	Population	Study design	Data collection instrument	Outcome measured	Result
Beckley (2007)	USA	95 Stroke survivors	Cross-sectional	Interviews	Community participation, Social Support, Functional limitation	Quality and quantity of social support played a significant role in participation.
Choi et al. (2015)	Korea	171 Stroke survivors	Cross-sectional	Surveys	Participation, depression, self-esteem, ADLs, social support	Psychological factors and ADLs directly affected participation.
Mayo et al. (2013)	Canada	102 Stroke survivors	Cohort	Surveys and objective tests	Participation, Mood, Social Support, Walking Capacity, Stroke Severity	The proportion of people with excellent or good social support showed excellent participation. Walking capacity influences participation.
Norlander et al. (2016)	Sweden	145 Stroke survivors	Cohort	Surveys	ADLs, depression, mental state, social and leisure activities	Driving, walking and extent of social network predicted positive outcomes.
Sumathipala et al. (2011)	UK	35 Stroke survivors	Qualitative	Semi-structured in depth interviews	Environmental (Physical, social and attitudinal) and personal factors	ICF environmental and personal factors including social support was viewed as a key facilitator of functioning.
Vincent-Onabajo et al. (2016)	Nigeria	96 Stroke survivors	Cross-sectional	Surveys	Participation, Social Support	Social support had correlations with overall participation, but was only significant in the self-sufficiency domain.

*Source*: Kumerenzi, A., Frantz, J., Rhoda, A. & Mlenzana, N., 2010, ‘Experiences of persons with physical disabilities regarding rehabilitation services, A systematic review,’ *Journal of Community and Health Sciences* 6(2), 33–39

ADL, activities of daily living.

### Quality assessment

The CASP appraisal tools for qualitative and cohort studies were utilised (Akobeng 2005; Critical Appraisal Skills Programme 1994), while the Milton Keynes Primary Trust for cross-sectional studies was used for the cross-sectional study (Milton Keynes Primary Trust 2002). All six articles were included in this review, as they obtained moderate-high scores for their quality assessment, representing a low risk of bias. The scores below represent the unanimous scores of both reviewers (see Table 2).

**TABLE 2:** Quality assessment scores.

Article	Q1	Q2	Q3	Q4	Q5	Q6	Q7	Q8	Q9	Q10	Q11	Q12	%	MA
Beckley (2007)	Y	Y	Y	N	Y	Y	Y	Y	Y	N	-	-	8	Y
Choi et al. (2015)	Y	Y	Y	Y	Y	Y	Y	Y	Y	N	-	-	9	Y
**Mayo et al. (2013)**	Y	Y	N	Y	N	Y	n/a	n/a	Y	c/t	c/t	n/a	6	Y
	-	-	-	-	N	Y	-	-	-	-	-	-	-	-
**Norlander et al. (2016)**	Y	Y	Y	Y	N	Y	n/a	n/a	Y	c/t	Y	n/a	8	Y
	-	-	-	-	N	Y	-	-	-	-	-	-	-	-
Sumathipala et al. (2011)	Y	Y	Y	Y	Y	N	Y	Y	Y	Y	-	-	9	Y
Vincent-Onabajo et al. (2016)	Y	Y	Y	Y	Y	c/t	Y	Y	Y	N	-	-	8	Y

Key: Q, Question; Y, Yes; N, No; n/a, no scoring required; c/t, cannot tell; %, percentage; MA, methodologically accepted.

### Demographic characteristics

An overview of the participant demographics for each article is tabulated below (see Table 3).

**TABLE 3:** Demographic characteristics.

Authors	Age	Gender	Employment at the time of stroke	Extent of functional limitation	Living condition
Beckley (2007)	68.46 ± 12.16	Majority female	Unknown	Majority functionally independent	Unknown
Choi et al. (2015)	53.67 ± 13.67	Majority male	Unknown	Unknown	Unknown
Mayo et al. (2013)	70.8 ± 13.1	Majority male	34% employed	Majority severely dependant on others	Unknown
Norlander et al. (2016)	Majority aged < 75 years	Majority male	Majority unemployed	Majority independent indoors and outdoors	Majority residing with partner or other(s)
Sumathipala et al. (2011)	69 ± 13.2	Majority female	Majority retired	Majority able to walk unaided	Majority residing with others
Vincent-Onabajo et al. (2016)	56.6 ± 12.0	Majority male	Majority unemployed	Unknown	Majority residing with family

### Outcome measures

Two of the studies identified utilised the ICF framework to categorise participation and social support and, as such, did not measure these variables specifically but made use of topic guides (Sumathipala et al. 2011) and measures of social and leisure activities (Norlander et al. 2016). The remaining articles included outcomes of both participation and social support. The four articles used different self-reported measures of participation. However, all measures comprised surveys in which participants were instructed to rate their responses on a 5-point Likert scale. This was performed at 3–6 months post-hospital discharge (Beckley 2007), 12 months post-stroke (Choi et al. 2015), and at 3, 6, 9 and 12 months post-stroke (Mayo et al. 2013). Vincent-Onabajo et al. (2016) measured participation in six domains, namely: mobility, physical independence, social integration, occupation, orientation and economic self-sufficiency. Although the articles utilised validated measures, there was a risk of response bias because these measures were self-reported. With regard to social support, Beckley (2007) measured the quality and quantity of social support received from family, friends, community individuals, community groups and professionals. Choi et al. (2015) measured emotional and informational support and Mayo et al. (2013) measured the extent of participants’ social network, while Vincent-Onabajo et al. (2016) measured the social support received from three sources, namely family, friends and significant others.

### Social support domains

#### Quality of social support

The quality of social support plays a significant role in participation (*p* = 0.03) at 3–6 months post-stroke, explaining 31% of the variance (*R*^2^ of 0.31) (Beckley 2007). Seventy-five per cent of participants gained emotional support from family and friends post-stroke, which played a vital role in participants’ functioning, thereby improving their participation (Sumathipala et al. 2011).

The instrumental support received from participants’ spouses or other family members was of assistance with ADLs. Subjectively, participants conveyed that they had always received more support from family and friends than was needed, even prior to the stroke (Beckley 2007). An elderly participant in the study conducted by Sumathipala et al. (2011) explained how the support she received from her family was not only practical, but lessened the pressure of managing her daily activities, which included providing her with transportation. This type of support aided her participation. She suffered a stroke 11 years ago, and still refers to these family members as ‘gems’. In this study, participants expressed that the support provided was more beneficial when it was based on need (Sumathipala et al. 2011).

### Quantity of social support

The quantity of social support plays a significant role in participation (*p* = 0.004) at 3–6 months post-stroke, explaining 35% of the variance (*R*^2^ of 0.35) (Beckley 2007). In this study, participation was the dependent variable and was measured by using a questionnaire asking participants how they managed in their homes, in the community, participating in meaningful and social activities and dealing with life events.

The extent of social networks had a significant effect on social and leisure activities 10 years post-stroke (*β* = 1.235; *p* = 0.004) (Norlander et al. 2016). Participants’ extensive support network can be explained by 93.1% having a particular person in their lives on whom they could depend, 63.4% engaging socially in the community every week, 33.3% having five different sources of social contact outside the household and 75.2% living with a partner or other(s). This was reported for the majority of participants and, as a result, social and leisure activities improved (*p* = 0.004). Of the three variables mentioned above, the number of sources of social contact was the only factor found to be significant (*r* = 0.369; *p* < 0.001) in predicting social and leisure activities.

Individuals who had high levels of social support prior to their stroke experienced greater social support initially post-stroke but, as time passed, a drop in social support levels was noticed, followed by a slow increase in support. Even after the drop and slow increase in social support, these individuals were still classified as having excellent social support, obtaining scores of above 80% (Mayo et al. 2013).

### The relationship between social support and participation

A recent study conducted in Nigeria found correlations between social support and overall participation (*p* < 0.05). Linear regression was applied and social support had a significant effect on the economic self-sufficiency domain of participation (*p* < 0.0001; *R*^2^ = 0.57). Social support had no significant and independent impact on overall participation in community-dwelling individuals post-stroke (*β* = 0.08; *R*^2^ = 0.57) (Vincent-Onabajo et al. 2016).

Sumathipala et al. (2011) reported that 74.0% of participants found that support from friends and family was a key facilitator towards functioning, which had shielded them from the impact of disability. In a few cases (8.0%) where support from family members was not guaranteed for the future, this resulted in poor participation. In addition, three participants had moved to houses that were closer to their friends and families to access the support they required to participate in activities.

Beckley (2007) found that as an individual’s independence decreases, their participation increases and the same applies to the reverse situation. As subjective social support increases, the estimate of functional limitation increases significantly (*p* = 0.003). A similar link was reported by Choi et al. (2015) where psychological factors mediated the relationship between social support and participation, that is, an increase in social support improved psychological well-being which positively affects participation. Psychological factors, as defined by the author, include depression, self-esteem and hopeful thinking. Therefore, social support had an indirect effect on participation post-stroke (*β* = -0.23; *p* = 0.01) via psychological factors (*β* = 0.50; *p* = 0.01).

In the process of determining the relationship between social support and participation post-stroke, Mayo et al. (2013) divided participants into categories based on the amounts of support they received. Social support was self-measured, which entailed five questions on the extent of participants’ social network. Participants (11.4%) scored support levels between 20 and 55 of the maximum (100) value and were classified as having poor social support, 52.4% of the sample scored between 60 and 70 of the maximum value and were classified as having fair social support, a further 26.4% of participants scored values of 80 and were classified as having very good social support, while the remaining 10.0% scored above 80.0% and were classified as having excellent social support. Fifty-six per cent of participants in the very good social support group had excellent levels of participation; a further 71.0% of the sample, classified as having poor social support, experienced poor participation.

## Discussion

The articles identified in this review stipulated distinct relationships between social support and participation where the quantity had a greater impact than the quality of support. This was a finding at 3–6 months post-stroke. It is important to consider the stage of recovery post-stroke. In the acute phase, individuals required large amounts of support to cope with the burden of disability, which would explain the above result (Beckley 2007). Individuals with limited support who needed to be able to return home post-stroke would then find the demands of returning to their pre-stroke roles challenging. It is important to note that social support as reported in this article is applicable to those individuals in community contexts.

The results demonstrate that the quality of support is generally provided over a long term, prior to the disability and maintained post-disability (Beckley 2007). The explanation for this can be threefold. Firstly, the presence of co-morbidities could be a confounding variable, which would explain why participants required support prior to the stroke. Participants in this review have been described as people with a primary diagnosis of stroke. The authors from the reviewed papers failed to mention participants’ medical histories, specifically with regard to co-morbidities. This cannot be overlooked as more than 50% of strokes in South Africa can be attributed to co-morbidities, including hypertension (Bertram et al. 2013). Secondly, the mean age of participants from the reviewed studies ranged from 53.67 to < 75 years. Older individuals have a greater need for social support which could lead to the need for care prior to the stroke. Social support provided prior to the stroke could have been related to the relationships within the specific families (Beckley 2007). Lastly, the support rendered might not have been based on need, as identified by Beckley (2007). The benefits of support provided were clearly highlighted by Sumathipala et al. (2011), because the ability of an individual to perform activities independently would aid in participation, more than having people in a person’s life that can assist with certain activities (Beckley 2007). This implies that the support provided should be based on the needs of the individual and dependent on the profile of the individual, concurrent with previous literature (Haun, Rittman & Sberna 2008).

It is suspected that individuals with close personal relationships receive more assistance than those without. This was reiterated by participants who expressed the amounts of support received from others (Beckley 2007). This echoes the extent of participants’ social support network, a topic that has been discussed in the literature (Haun et al. 2008). Maintaining a strong social support system has been found crucial to improving quality of life (QoL) post-stroke (Boden-Albala et al. 2005; Glass & Maddox 1992). More specifically, at 10 years post-stroke, the quantity of social support and extent of support networks are directly linked to positive outcomes in participation, a finding from this review (Norlander et al. 2016). In addition, recent literature has revealed that an extensive support network aids return to work (RTW) post-stroke (Wang, Kapellusch & Garg 2014). A very small proportion of participants (34%) was employed at the time of their stroke. In South Africa, there is minimal literature available on RTW intervention platforms for individuals with stroke (Ntsiea et al. 2015), so this information could be useful to plan rehabilitation strategies to facilitate RTW. An interesting finding was observed in the study by Mayo et al. (2013), which reiterated the importance of an extensive support network. The inconsistencies in participants’ support noted, after the initial phases, could have affected their participation in a negative way had it not been for the large amounts of support received.

The studies conducted by Beckley (2007) and Choi et al. (2015) show the indirect effect that social support has on participation via other variables. This demonstrates the profound effect that physical impairment and depression has on participation, a finding in line with previous literature (Maleka et al. 2012; Mayo et al. 2013). Vincent-Onabajo et al. (2016) found that higher levels of social support were linked to better participation in relation to economic self-sufficiency. To be economically self-sufficient entails the maintenance of income to achieve basic needs. The authors suspect that this result was achieved because support was being rendered financially. This could be linked to the low rate of occupational participation in the study, a result found in a number of reviewed studies as well. The mean age of participants across the studies also needs to be taken into consideration, as the majority of participants in the studies could be retirees.

The remaining studies provide evidence on the direct effect of social support on participation post-stroke, where high levels of social support improve participation (Beckley 2007; Mayo et al. 2013). The same applies to the reverse situation, where participants who experienced limited support from family reported difficulties with participation (Sumathipala et al. 2011). These results are seen up to 10 years post-stroke, as identified by a cohort (Norlander et al. 2016). A strength identified by Sumathipala et al. (2011) and Norlander et al. (2016) was using the ICF as a framework to examine contextual factors related to stroke. These studies have also contributed significantly to the development of the ICF. Although Mayo et al. (2013) and Norlander et al. (2016) utilised the same study design, their methodologies were different and, as a result, analysing them together was challenging. The studies identified cannot be generalised because of the small sample size and excluding participants with severe cognitive defects. A recent South African study found that 46% of participants reported mild-moderate cognitive impairment post-stroke, which affected their QoL (Arowoiya et al. 2017). Cognitive impairments have also been found to influence participation in leisure activities and employment (Pinquart & Sorensen 2006). In their attempt to include participants with cognitive impairment, Norlander et al. (2016) utilised proxy respondents, which could have affected the results. At the other end of the spectrum, these results could be generalised to the cognitively impaired population.

## Conclusion

This review produced six articles that showed significant relationships between social support and participation post-stroke. Important aspects to consider with regard to social support are the quality, quantity and timing of support. The results illustrate that for the quantity of social support to have a significant effect on participation, the support needs to be established prior to the stroke. This support would be beneficial if it was provided in generous quantities so that when there is a decrease in support levels after the stroke, the individual would only be mildly affected. A finding from this review is that for the quality of social support to have positive outcomes on participation post-stroke, it needs to be based on the requirements of the individual concerned, who values the emotional and instrumental support received. This review further highlighted the influence of physical impairment and altered mental status on participation, as well as RTW post-stroke. The ICF framework has been found to be effective in analysing participation restrictions and environmental factors linked to social support.

### Implications for practice

It is clear that social support is a vital factor to consider when managing the individual with stroke holistically, which includes planning rehabilitation interventions. This information is particularly important to Allied Health Professionals working in the clinical setting. A theme that emerged from this review was the importance of an individual’s quantities of support and the extent of support networks. To address this, rehabilitation strategies and interventions could focus on incorporating group activities. Social support interventions would aid the re-integration of individuals back into the community. Interventions should include group sessions with family members and caregivers, where the focus should be on assisting individuals to gain independence. Outdoor activities with support structures should be encouraged, to aid social support and participation in the community.

A finding from this review is that physical impairment is significantly related to reduced participation. It is vital for healthcare policies to consider community access support and mobility aids, including the provision of transport for individuals with disabilities, to allow them to function optimally in the community. These should be easily accessible and affordable. Future studies should be conducted in the form of RCTs, as none were identified on the topic in question. Social support should be measured as a multidimensional concept to include all aspects.

### Limitations of the study

This review is not a complete representation of the available literature, as only English-text articles were used from distinct databases at a single institution; thus, publication bias could be present. Only self-reported measured were utilised as outcomes in the reviewed studies which could present response bias. Another limitation was that no RCTs were found resulting in the inclusion of lower levels of evidence. In addition, all types of study designs were utilised, making the comparison of articles a challenging exercise.

## Appendix 1

**TABLE 1-A1:** PICO analysis.

Article	Title	P (opulation)	I (ntervention)	C (omparison)	O (utcome)	Accept/Reject	Reasons
1							
↓							
54							

## Appendix 2

**TABLE 1-A2:** Quality assessment tool for cross-sectional studies.

Question	Description
1	Did the study address a clearly focused question?
2	Did the authors use an appropriate method to answer their question?
3	Were the subjects recruited in an acceptable way?
4	Were the measures accurately measured to reduce bias?
5	Were the data collected in a way that addressed the research issue
6	Did the study have sufficient participants?
7	How are the results presented?
8	Was the data analysis sufficiently rigorous?
9	Is there a clear statement of findings?
10	Can the results be applied to the local population?

*Source*: Keynes Primary Trust 2002
